# Birth defects: etiology to prevention

**DOI:** 10.1186/1755-8166-7-S1-I24

**Published:** 2014-01-21

**Authors:** Koumudi Godbole

**Affiliations:** 1 Deenanath Mangeshkar Hospital and Research Center, Erandawane, Pune, India

## 

Structural birth defects together of are a prominent cause of mortality and morbidity and are gaining importance with improving obstetric care and reduction in infective causes. Advances in understanding genetic (G) and environmental (E) factors and their interaction have added newer dimensions to the etiology of birth defects. Epigenetic is one such interesting field expected to provide mechanistic links between whether and how the environment interacts with maternal and fetal genomes. Some classical examples of environmental insults such as teratogens, maternal medical disorders and nutritional deficiencies as well as G-E interactions leading to birth defects will be discussed. It is important that the relevant scientific information should reach professionals and public for better awareness, appropriate policy decisions to prevent birth defects.

